# Oral-microbiome-derived signatures enable non-invasive diagnosis of laryngeal cancers

**DOI:** 10.1186/s12967-023-04285-2

**Published:** 2023-07-05

**Authors:** Shuting Yu, Junru Chen, Yan Zhao, Fangxu Yan, Yue Fan, Xin Xia, Guangliang Shan, Peng Zhang, Xingming Chen

**Affiliations:** 1 Department of Otolaryngology-Head and Neck Surgery, Peking Union Medical College Hospital, Peking Union Medical College and Chinese Academy of Medical Sciences, 1 Shuaifuyuan, Dongcheng District, Beijing, China; 2 Institute of Chinese Medical Sciences, University of Macau, Taipa, Macao China; 3 Department of Otolaryngology Head and Neck Surgery, Beijing TongRen Hospital, Capital Medical University, Beijing, China; 4 Department of Epidemiology and Statistics, Institute of Basic Medical Sciences, Chinese Academy of Medical Sciences and School of Basic Medicine, Peking Union Medical College, Beijing, China; 5 Beijing Key Laboratory for Genetics of Birth Defects, Beijing Pediatric Research Institute, MOE Key Laboratory of Major Diseases in Children, Rare Disease Center, Beijing Children’s Hospital, Capital Medical University, National Center for Children’s Health, Beijing, China

**Keywords:** Biomarker, Microbiota, Laryngeal cancer, Diagnosis, Liquid biopsy

## Abstract

**Background:**

Recent studies have uncovered that the microbiota in patients with head and neck cancers is significantly altered and may drive cancer development. However, there is limited data to explore the unique microbiota of laryngeal squamous cell carcinoma (LSCC), and little is known regarding whether the oral microbiota can be utilized as an early diagnostic biomarker.

**Methods:**

Using 16S rRNA gene sequencing, we characterized the microbiome of oral rinse and tissue samples from 77 patients with LSCC and 76 control patients with vocal polyps, and then performed bioinformatic analyses to identify taxonomic groups associated with clinicopathologic features.

**Results:**

Multiple bacterial genera exhibited significant differences in relative abundance when stratifying by histologic and tissue type. By exploiting the distinct microbial abundance and identifying the tumor-associated microbiota taxa between patients of LSCC and vocal polyps, we developed a predictive classifier by using rinse microbiota as key features for the diagnosis of LSCC with 85.7% accuracy.

**Conclusion:**

This is the first evidence of taxonomical features based on the oral rinse microbiome that could diagnose LSCC. Our results revealed the oral rinse microbiome is an understudied source of clinical variation and represents a potential non-evasive biomarker of LSCC.

**Supplementary Information:**

The online version contains supplementary material available at 10.1186/s12967-023-04285-2.

## Introduction

The human body harbors trillions of microorganisms with various functions that are closely related to our health. Being one of the largest habitats of microorganisms, there are more than 1000 different kinds of microorganisms in the oral cavity. Plenty of studies showed that altered oral microbial profile and their metabolites are associated with head and neck squamous cell carcinoma (HNSCC) [1, 2] and esophageal cancer [3], and they might also influence remote organs through the digestive tract [4], thus contributing to colorectal cancer [5], pancreatic cancer [6], and lung cancer [7]. Through extensive studies, some specific taxa were identified to be related to oral/oropharyngeal cancers, such as *Fusobacterium*, *Peptostreptococcus*, *Prevotella*, *Veillonella*, *Capnocytophaga*, etc. Several mechanisms of carcinogenic action of oral bacteria have been proposed [8–10]. The first is bacterial stimulation of chronic inflammation. Anaerobic species such as *Fusobacterium*, *Prevotella*, and *Porphyromonas* are responsible for periodontal diseases and lead to chronic inflammation that may facilitate cell proliferation, angiogenesis, and oncogene activation. The second mechanism involves antiapoptotic activity. Bacteria such as *Porphyromonas gingivalis* inhibit apoptosis by modulating several pathways [11]. The third mechanism is the direct or indirect production of carcinogenic substances. Several oral bacteria are capable of metabolizing alcohol to carcinogenic acetaldehyde [12]. Besides, genotoxic metabolites of certain bacteria could cause DNA damage or produce free radicals and affect reactive oxygen species (ROS) [13, 14].

Laryngeal squamous cell carcinoma (LSCC) is the second most prevalent in all respiratory system carcinomas after lung cancer [15]. LSCC is also common among HNSCC. Approximately 184,615 new cases and 99,840 deaths were estimated attributing to LSCC worldwide in 2020 [15]. However, due to the lack of early detection, most patients have developed advanced LSCC upon diagnosis [16, 17], which always leads to regional recurrence and distant metastase even after surgical resection. The pathogenesis of LSCC is not fully uncovered, and several risk factors are related to LSCC, such as smoking, alcohol, and human papillomavirus (HPV) infection.

Recently, several studies of head and neck cancer showed that alterations in microbiota may drive HNSCCs, and some potentially oncogenic bacteria are identified [9, 18, 19]. However, prior work investigating the microbiome of head and neck cancer mainly focused on oral/oropharyngeal cancer. There is very limited data to explore the unique microbiota of laryngeal cancer, in addition, whether the oral microbiome could serve as a biomarker for the non-invasive early detection of laryngeal cancer has not been studied. The oral microbiome from oral rinse has been considered as a good tool to study the microbiota of oral/oropharyngeal cancer due to the close anatomical sites [1, 20]. It is also a promising biomarker for the early detection of cancer with lower cost and noninvasive access. As the oral microbiota can migrate to the laryngopharyngeal region through inhalation and swallowing, this study aimed to identify alterations in the microbiome of LSCC patients from control-group patients with tissue samples and oral rinse samples, and explore whether oral microbiota can be applied as non-evasive diagnostic markers in LSCC patients.

## Materials and methods

### Clinical samples and database

Patients were enrolled in this study at Peking Union Medical College Hospital (PUMCH) from 2020 to 2022. All the patients were divided into two groups: (1) tumor group, 77 patients who had pathologically confirmed, previously untreated LSCC undergoing radical resection; (2) control group, which was composed of 76 patients who had vocal cord polyps matched by age and gender. The exclusion criteria were as follows: (i) antibiotics therapy in a month; (ii) infection with HBV, HCV, syphilis, or HIV; (iii) a history of malignant tumors, chemotherapy, or radiotherapy. TNM stages of all participants were identified according to National Comprehensive Cancer Network (NCCN) Guidelines in 2021. Clinical information such as age, sex, tobacco, and alcohol consumption was obtained from the medical history of the participants. “Smokers” were defined as patients who had smoked > 100 cigarettes during their lifetime; otherwise, they were defined as “never smokers”. “Drinkers” were defined as those who drank alcohol at least once a week for a year or longer; whereas “never drinkers” had drunk less. Before sampling, participants were banned from dieting, smoking, and oral hygiene prophylaxis for at least 2 h. Participants rinsed the mouth vigorously with 10 ml sterile saline for 30 s in the operating room to sterilely collect oral rinses. Then, cancerous tissues or polyp tissues from the central area of the lesions were obtained in the surgery. Tissue samples were placed into sterile 2 ml Eppendorf tubes (Axygen, USA) and then frozen at − 80 degrees Celsius before further processing.

### 16S rRNA sequencing data analysis

Total genomic DNA was extracted from the specimens using the QIAampFast DNA Stool Mini Kit (Qiagen, Hilden, Germany). The V3-V4 region of the bacterial 16S rRNA gene was amplified with PCR using the forward primer 341F (5′-barcode-CCTAYGGGRBGCASCAG-3′) and reverse primer 806R (5′-GGACTACNNGGGTATCTAAT-3′). PCR was performed in a mixture of 30 μl as previously described. Pooled PCR products were extracted using 2% agarose gels and then purified using a GeneJET Gel Extraction Kit (Thermo Fisher Scientific). NEB Next^®^ Ultra™ DNA Library Prep Kit (New England Biolabs) was applied for generation of the sequencing libraries, and the index codes were added. The qualified library was sequenced on an Illumina NovaSeq platform, and 250 bp paired-end reads were generated according to the standard protocols.

### Bioinformatics analysis

Paired-end reads were merged into tags with FLASH (version 1.2.7) [21]. Raw tags were filtered using the command split_libraries_fastq.py in QIIME (version 1.8) [22]. Chimeric tags were removed with USEARCH (version 6.1) [23] after quality control. The satisfied reads were clustered into operational taxonomic units (OTUs) at 97% similarity using QIIME. Taxonomic information on OTUs was obtained with the Ribosomal Database Project classifier [24] against the Greengenes database (release 13.8) [25]. Alpha diversity indices and β-diversity distance matrices measuring the pairwise difference among samples were calculated with QIIME. Differences in the Chao1 index, Shannon index, Simpson index, and observed OTUs were detected using the Wilcoxon rank sum test. Principal coordinates analysis (PCoA) was conducted using R software (version 4.0.2) [26], PERMANOVA was performed with the vegan [27] package in R to clarify differences in microbial communities between groups. The Wilcoxon rank sum test was applied to identify differentially abundant taxa between groups, and a false discovery rate < 0.05 was considered to be statistically significant.

Using genus profiles, samples were randomly divided into a training set and a test set by using the “sample” function (80% of samples as training dataset and 20% of samples as testing dataset). Only genera that were present in at least 10% of subjects were considered in the analyses. The number of selected biomarkers were determined after tenfold cross-validation that was repeated for five times based on the training set profile. Then, the final random forest classification models were constructed, and the mean decrease accuracy of selected markers were used to evaluate the importance of biomarkers. The area under the curve (AUC) was used to measure the performance of the models when applied to the training and test sets using the ROCR (version 1.0–11) [28] package in R, and receiver-operating characteristic (ROC) curves were calculated using the pROC (version 1.17.0.1) [29] package in R. The code for bioinformatics analysis can be requested at https://github.com/Jora1991/Oral-microbiome-for-laryngeal-cancers.

## Results

### Diversity dysbiosis of the tumor and oral microbiome in LSCC

**Fig. 1:**
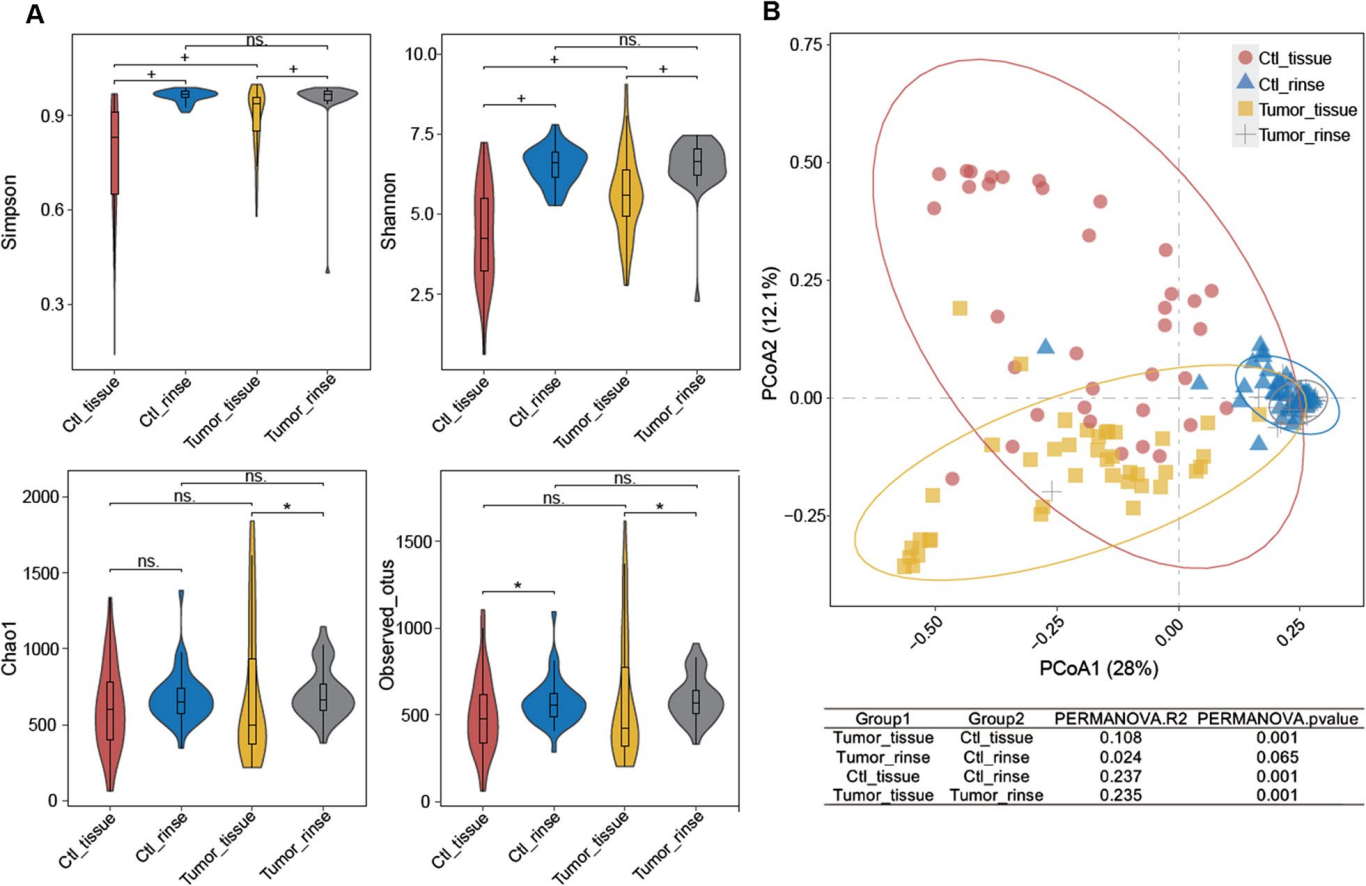
Microbiota diversity in LSCC (N = 77) and control samples (N = 76). **A**. Comparison of microbial α-diversity between groups. P values were calculated from the Wilcoxon rank sum test. P < 0.01 was labeled with “+”, and “*” represented P < 0.05. **B** Principal coordinate analysis (PCoA) plots. The P value was derived from permutational multivariate analysis of variance (PERMANOVA)

A total of 153 patients were enrolled in this study at Peking Union Medical College Hospital (PUMCH) from 2020 to 2022. Most patients were between 56 and 70 years old (66.0%), and average age of patients was 60.4. The male/female ratio was 8.6:1 (137 males:16 females). According to cancer statistics, laryngeal cancer occurs more commonly in men than in women, most frequently diagnosed among people aged 55–74 [15, 30]. The male/female ratio and age range of LSCC patients in this study approximately corresponded with the epidemiology of laryngeal cancer in literature. The baseline characteristics of the entire cohort are presented in Table 1. As is shown, no significant differences were found between the LSCC group and the control group regarding age or gender. Smokers and drinkers were more common in the LSCC group, which was inevitable. Community alpha diversity was assessed with observed taxonomic units (OTUs), Simpson, Shannon, and Chao1 indices (Fig. 1A). Statistical *P* values of alpha diversity indices among different groups are presented in Additional file 1: Table S1. The oral rinse samples exhibited more elevated microbial diversity indices compared with the tissue samples in both the tumor group and control group (P value < 0.05 for the Simpson index, Shannon index, and observed OTUs). The Simpson index and Shannon index were significantly higher in the tumor tissues than in the control tissues. Although the oral rinse samples did not show significant differences in α-diversity between the tumor and control group, it is worth noting that the in-group variation of oral rinse samples was lower than that of tissue samples. Then, principal coordinate analysis (PCoA) was performed based on the Bray–Curtis distance matrix from the genus profile to investigate β-diversity. As shown in Fig. 1B, the oral rinse samples were clustered closely with each other and separated from the tissue samples, in which a clear distinction between tumor tissue and control tissue samples was found based on permutational multivariate analysis of variance (PERMANOVA). In summary, oral rinse samples presented lower within-group variation compared to laryngeal tissue samples.

**Table 1:** Demographics and clinical characteristics of the study cohort

	Entire cohort (N = 153)					Train set (N = 121)					Test set (N = 32)				
	LSCC (N = 77)		Control (N = 76)		P	LSCC (N = 64)		Control (N = 57)		P	LSCC (N = 13)		Control (N = 19)		P
	No	%	No	%		No	%	No	%		No	%	No	%	
Age (y)															
≤ 55	12	15.6	21	27.6	0.182	10	15.6	16	28.1	0.242	2	15.4	5	26.3	0.737
56–70	54	70.1	47	61.8		45	70.3	35	61.4		9	69.2	12	63.2	
> 70	11	14.3	8	10.5		9	14.1	6	10.5		2	15.4	2	10.5	
Gender (%)															
Male	71	92.2	66	86.8	0.278	59	92.2	50	87.7	0.412	12	92.3	16	84.2	0.496
Female	6	7.8	10	13.2		5	7.8	7	12.3		1	7.7	3	15.8	
Smoking status															
Smokers	66	85.7	27	35.5	< 0.01	56	87.5	21	36.8	< 0.01	10	76.9	6	31.6	0.012
Nonsmokers	11	14.3	49	64.5		8	12.5	36	63.2		3	23.1	13	68.4	
Alcohol															
Drinkers	45	58.4	19	25.0	< 0.01	39	60.9	16	28.1	< 0.01	6	46.2	3	15.8	0.061
Nondrinkers	32	41.6	57	75.0		25	39.1	41	71.9		7	53.8	16	84.2

### Microbial composition alterations of LSCC tumor and oral rinse samples

**Fig. 2:**
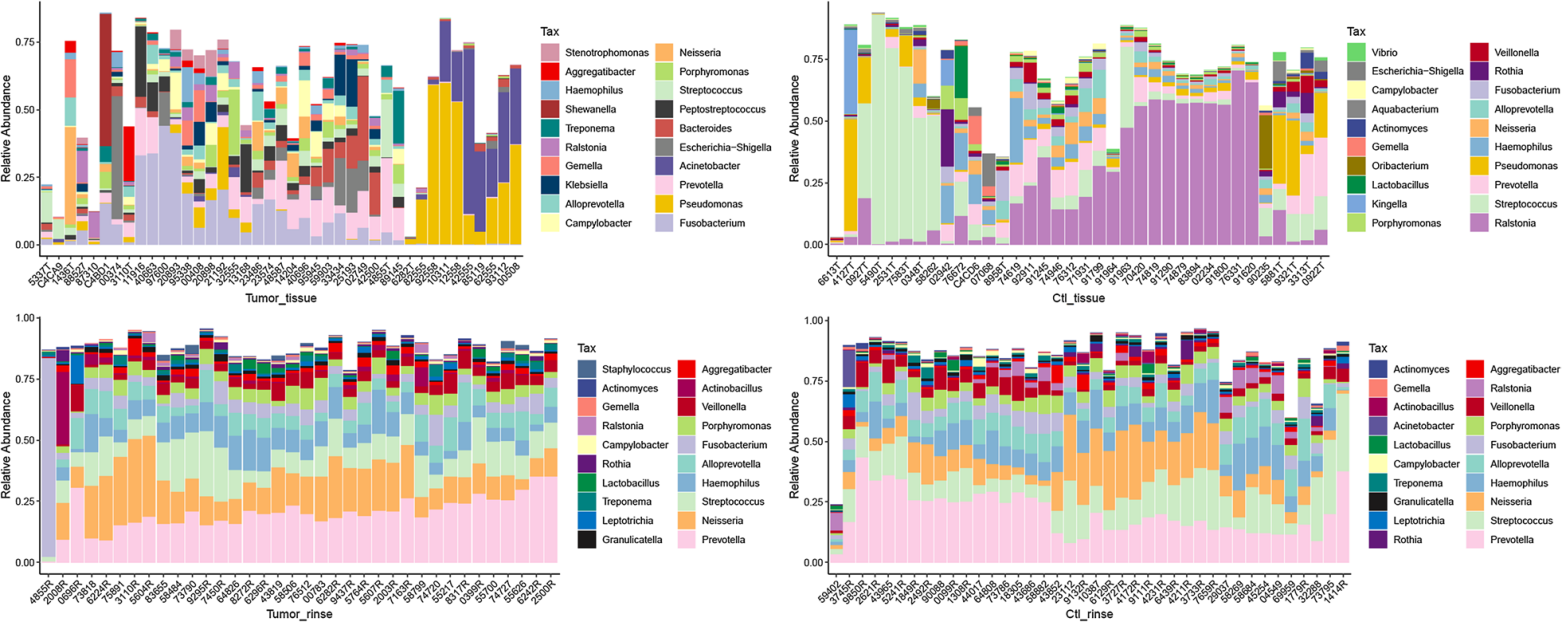
Dominant genera in LSCC (N = 77) and control samples (N = 76). The relative abundance of the top 20 dominant genera in different groups were presented. Samples of each group were clustered and ordered based on the abundance profile of top 20 genera using “hclust” function in R

**Fig. 3:**
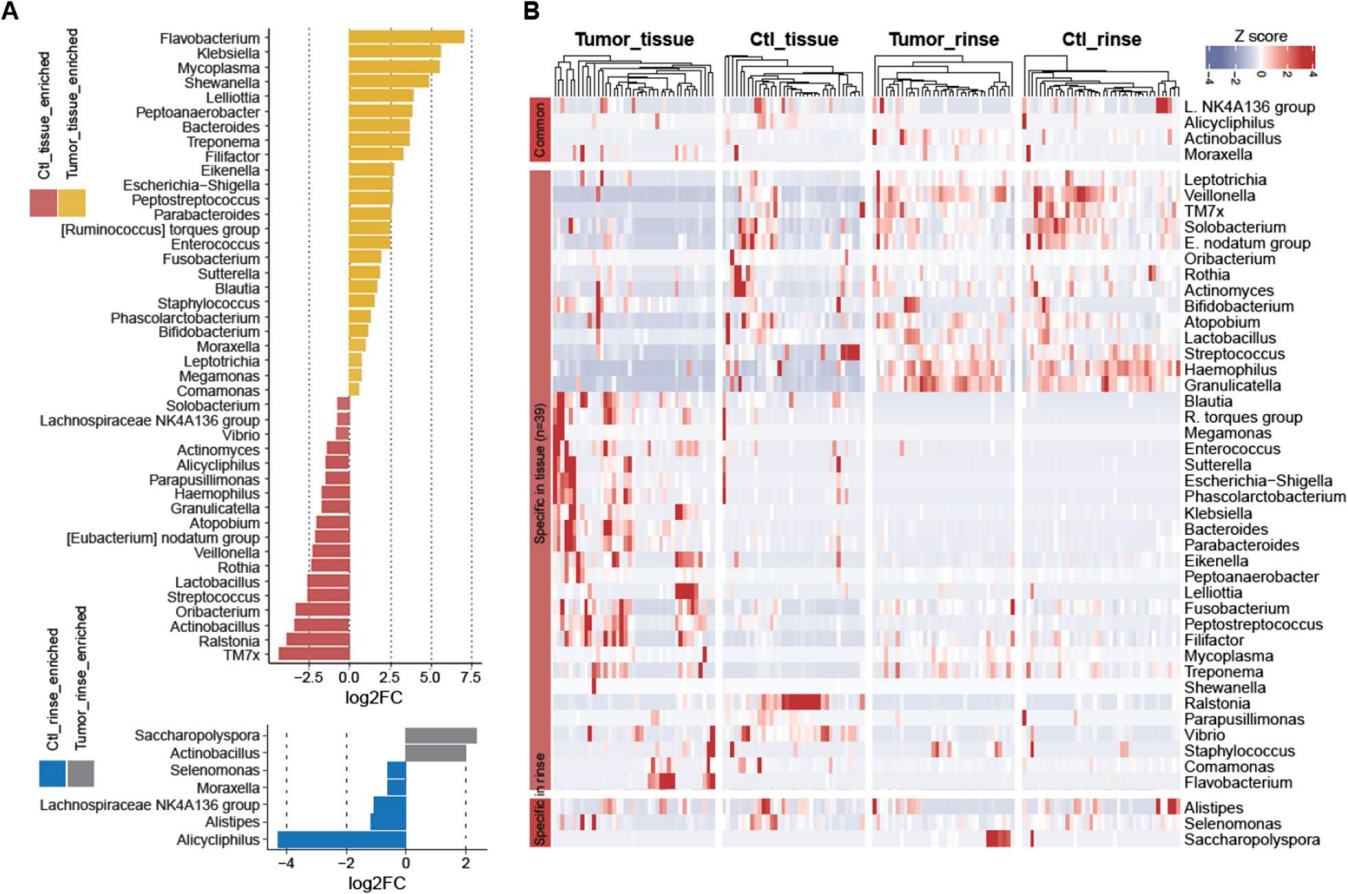
Differential microbial composition in LSCC tumor tissues and oral rinse samples. **A** Log2-fold change (Log2FC) of the genera was significantly different between tumor tissue and control tissue samples (above panel); Log2FC of the genera was significantly different between oral rinse samples from tumor patients and oral rinse samples from control patients (lower panel). Log2FC > 0 indicated that the genus was more abundant in tumor patients than in control patients, while log2FC < 0 indicated that the genus was less abundant in tumor patients than in control patients. **B** Colored heatmap according to the abundances of differential genera in **A**

Subsequently, the relative abundance of the top 20 taxa was compared between specimens from LSCC patients and specimens from control patients (Fig. 2). At the genus level, tumor tissues contained a much higher percentage of *Fusobacterium* (0.088 ± 0.115 (mean ± SD) vs. 0.022 ± 0.028, tumor vs. control tissues)*, Pseudomonas* (0.083 ± 0.160 vs. 0.055 ± 0.101)*,* and *Acinetobacter* (0.055 ± 0.127 vs. 0.003 ± 0.013) compared to control tissues, whereas *Ralstonia* (0.016 ± 0.026 vs. 0.233 ± 0.241) were prominent constituents of most control tissues, but were scarce in tumor tissues. Oral rinse samples displayed similar microbiome composition and were characterized by the same general in both the tumor group and control group (*Prevotella*, *Neisseria*, *Streptococcus*, *Haemophilus*, and *Alloprevotella*), which also implied lower within-group variation compared to laryngeal tissue samples. As expected, significant differences in the microbiota composition at the genus level were discovered among different groups of samples (Fig. 3A). Compared with the control tissues, the tumor tissues exhibited a marked increase in the abundance of several genera, such as *Flavobacterium* (*P* = 0.008, 0.003 ± 0.007 vs. 0.000 ± 0.000, tumor vs. control tissues), *Klebsiella* (*P* = 0.002, 0.017 ± 0.038 vs. 0.000 ± 0.001), *Mycoplasma* (*P* = 0.001, 0.002 ± 0.011 vs. 0.000 ± 0.000), etc., while the abundances of *Ralstonia* (*P* = 0.000, 0.012 ± 0.026 vs. 0.233 ± 0.241, tumor vs. control tissues), *Actinobacillus* (*P* = 0.000, 0.000 ± 0.000 vs. 0.002 ± 0.005), *Streptococcus* (*P* = 0.000, 0.024 ± 0.039 vs. 0.151 ± 0.238), *Lactobacillus* (*P* = 0.000, 0.002 ± 0.006 vs. 0.010 ± 0.035), *Rothia* (*P* = 0.002, 0.003 ± 0.006 vs. 0.015 ± 0.041), and several other genera were significantly lower in the tumor tissues. In the oral rinse samples, *Saccharopolyspora* (*P* = 0.018, 0.001 ± 0.003 vs. 0.000 ± 0.002, tumor vs. control rinses) and *Actinobacillus* (*P* = 0.008, 0.019 ± 0.050 vs. 0.005 ± 0.010) were more abundant from tumor patients than those from control patients. The heatmap is colored according to the relative abundances (Fig. 3B) showing the genera differences among the four sample groups (the tumor tissue samples, the control tissue samples, the oral rinse samples from LSCC patients, and the oral rinse samples from control patients). We found that the tissue samples were separated from the oral rinse samples and that the tumor tissue samples were distinct from the control tissue samples. The genera *Veillonella*, *Fusobacterium*, *Ralstonia*, etc., contributed to this separation. Additionally, 39 genera were observed only in the differential analysis of the tissue samples (*Leptotrichia, Veillonella, Solobacterium,* etc.; shown in Fig. 3B). Three unique genera, *Alistipes, Selenomonas, and Saccharopolysora* were observed only in the differential analysis of the oral rinse samples. Four genera, *L.NK4A136, Alicycliphilus, Actinobacillus, and Moraxella* were detected in the differential analysis of both the oral rinse and tissue samples. More details about the percentage values and statistical *P* values of genera among different groups are presented in Additional file 1: Table S2.

### Classification model for LSCC diagnosis based on oral rinse microbiota

**Fig. 4:**
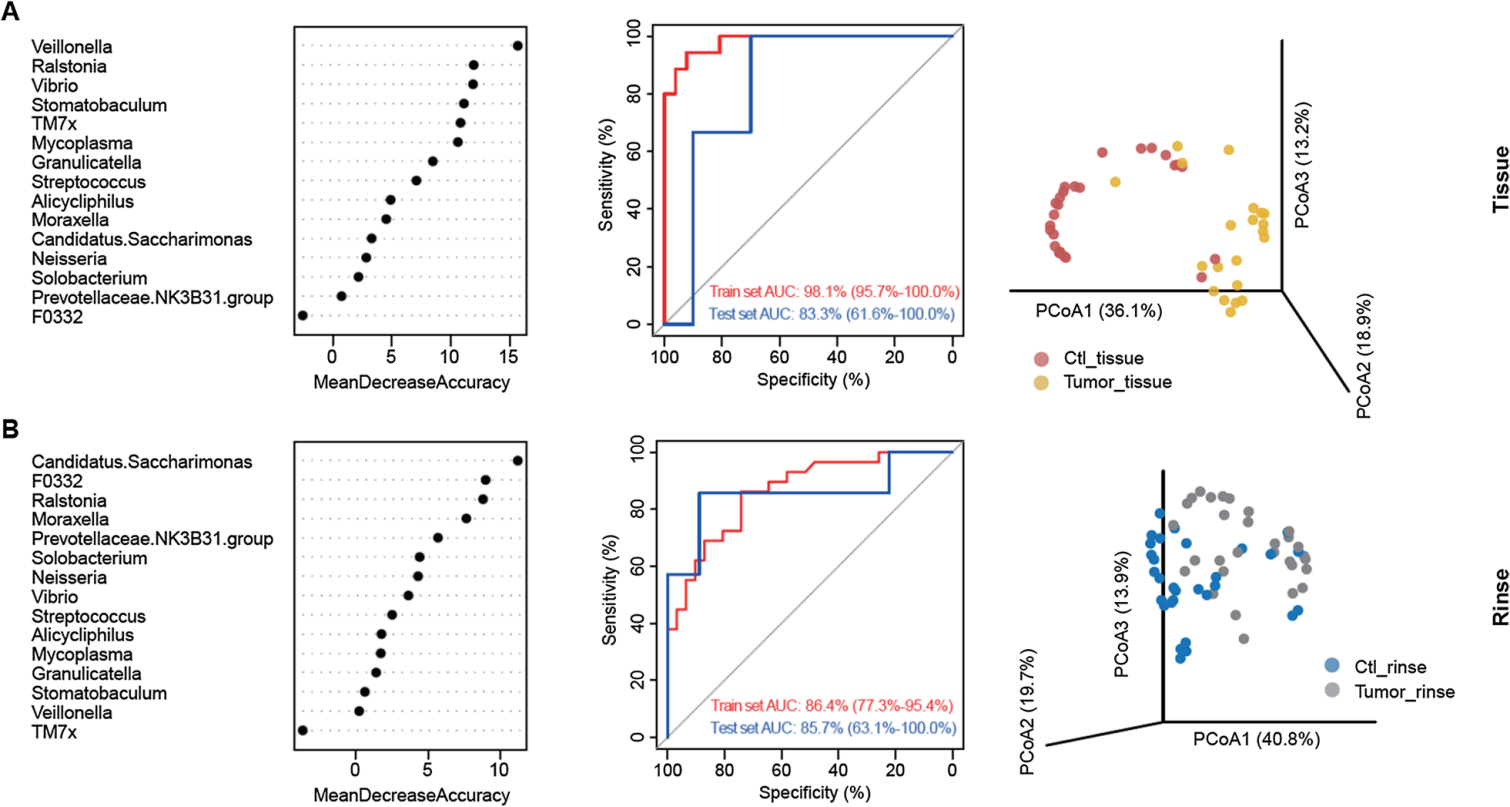
Identification of microbial-based markers for clinical diagnosis by random forest models.** A** Mean decrease accuracy (MDA) of variables from the RF model using the profile of 15 selected biomarkers in tissue samples (left). ROC curves are based on the training set and test set from tissue samples (middle). The AUC is displayed in the lower right-hand corner. PCoA based on the profile of 15 selected genera in tissue samples (right). The P value is derived from PERMANOVA. **B** MDA of 15 selected biomarkers (left), ROC curves, and AUC (middle) based on the RF model from oral rinse samples. PCoA and PERMANOVA based on the profile of 15 selected genera in oral rinse samples (right)

Our results suggested that the abundance of several taxa was significantly different in the oral rinse samples between the LSCC group and the control group. Therefore, the oral microbiota has the potential to be a predictive marker indicating a risk for LSCC. To test this hypothesis, microbial biomarkers were selected first at the genus level using tenfold cross-validation that was repeated five times based on a training set of 121 samples (Table 1). Then, two random forest (RF) models were trained from the selected biomarkers utilizing the training sets from tissue samples (N = 61) and oral rinse samples (N = 60). The performances of the constructed models were evaluated by the area under the curve (AUC) of receiver operating characteristic (ROC) curves. A total of 15 genera, including *Candidatus Saccharimonas*, *Ralstonia*, *Moraxella*, *Solobacterium*, *Neisseria*, *Streptococcus*, *Veillonella,* and 8 others, were identified as microbial biomarkers that could discriminate LSCC patients from control patients in both tissue samples (Fig. 4A) and oral rinse samples (Fig. 4B), indicating the potential use of microbial features in the identification of LSCC. Specifically, the model for tissue samples exhibited excellent performance based on the training set (AUC = 98.1%) and the test set (N = 16, AUC = 83.3%) (Fig. 4A). Interestingly, compared with the previous model using tissue samples, the RF model for oral rinse samples showed a higher AUC based on the test set (N = 16, AUC = 85.7%) accompanied by a lower AUC based on the training set (AUC = 86.4%) (Fig. 4B), supporting the potential diagnostic value of our oral microbiota-based classifier for LSCC. Moreover, we also examined the potential ability of the 15 microbial biomarkers to discriminate LSCC based on PCoA and PERMANOVA. Significant separations (PERMANOVA, P < 0.05) between the LSCC and control groups were observed.

## Discussion

While the role of oral microbiota in oral/oropharyngeal cancer has received much attention, few data in the literature investigated the association between microbial alteration and laryngeal cancer [31–33]. None of the studies included both oral rinse/saliva samples and tissue samples to study the alterations of oral microbiota and laryngeal microbial composition in LSCC patients. The present study included both sample types to investigate whether oral rinse samples can replace tissue samples in microbiome studies and be applied as early diagnostic markers in LSCC patients. Our study showed that although oral rinse samples exhibited remarkably elevated microbial diversity indices compared with the tissue samples, and the oral rinse samples did not show significant differences in α-diversity or β-diversity between the tumor and control group, oral rinse samples presented lower within-group variation compared with laryngeal tissue samples, which indicated their potential character as a stable diagnostic biomarker. Compared with the control tissues, the abundance of several genera was significantly higher in the laryngeal tissue of the LSCC group, including some notorious genera such as Fusobacterium, which is in accordance with previous studies [31, 34]. Fusobacterium is an invasive anaerobe mainly associated with periodontitis, which may lead to chronic inflammation and contribute to carcinogenesis. Recently, more and more studies have found an association between the abundance of fusobacterium and cancer, including oral/ oropharyngeal cancer [35], colorectal cancer [36, 37], and so on. In the oral rinse samples, taxa differences between the tumor group and the control group were not as large as those based on the tissue samples, however, the RF model using microbial biomarkers from oral rinse samples exhibited excellent performance based on the training set and the test set, indicating the diagnostic value of our oral microbiota-based classifier for LSCC.

Of note, no previous study had proven that a microbiota-based model could reliably detect laryngeal cancer through oral rinse samples with high sensitivity and specificity. We identified taxonomic features that yielded a model and developed a classifier, which was able to distinguish cancer status. Our results demonstrate that oral microbiome shifts might serve as a marker in advance of the clinical phenotypes detectable by endoscope or CT scans. As this study describes cohorts of patients with diagnosed laryngeal cancer, future work should examine if this signature can be applied to assess cancer onset in individuals with precancerous lesions such as vocal leukoplakia. This is a good clinical endpoint as the model enables non-invasive LSCC detection with a simple taxa quantification test on oral rinse samples. Our findings provide a strong rationale for developing microbiome-based liquid biopsy technology to prioritize at-risk individuals for clinical attention.

Although this is not the pilot study to reveal the specific microbial composition of LSCC patients, our study is the first one to include multiple sample types and investigate whether oral rinse samples can replace tissue samples in microbiome studies. Several unavoidable limitations inherent in this study should be acknowledged. First, this was a retrospective study conducted in a single institution and therefore involved possible selection bias. Second, although age and gender are paired in two groups, the influence of smoking and alcohol consumption on microbiota cannot be eliminated in this study. Tabbco and alcohol consumption could alter oral microbial composition [9, 38], and might lead to the natural selection of microbiota capable of a high rate of carcinogen metabolism, which may synergize with the primary risk factors such as alcohol abuse and smoking in cancer pathogenesis [39–41]. In the present study, there are no genera differences in oral rinses between smokers and non-smokers of control group, and only one genus (Ralstonia) was found in microbial differential analysis in oral rinses between drinkers and non-drinkers of control group. In brief, we believe that smoking and drinking showed limited influence on microbiota from oral rinses in this study, so the classification model for LSCC diagnosis based on microbiota from oral rinses is solid. Further studies conducted with more patients will be performed to allow sub-group analysis.

## Conclusion

Through the analysis of alpha and beta diversity, we found that oral rinse samples presented lower within-group variation compared with laryngeal tissue samples, which indicated their potential character as a stable diagnostic biomarker. Then, our results proved that dysbiosis of the oral microbiome is a key hallmark of LSCC, and the oral microbiota-based model exhibited excellent performance in the discrimination of LSCC samples. To our knowledge, this is the first study to provide a strategy for the non-invasive prediction of LSCC by employing an oral microbiota-based model, which represents a promising early diagnostic biomarker of LSCC patients for future clinical use.

## Supplementary Information

**Additional file 1: Table S1.** Statistical P values of alpha diversity indices among different groups. **Table S2.** Percentage values and statistical P values of genera among different groups.

## Data Availability

The datasets used for the current study are available upon reasonable request from the corresponding author.

## Abbreviations

LSCC: Laryngeal squamous cell carcinoma
HNSCC: Head and neck squamous cell carcinoma
OTU: Observed taxonomic units
RF: Random forest
AUC: Area under the curve
ROC: Receiver operating characteristic curves
PCoA: Principal coordinate analysis
